# Type I Interferon Protects against Pneumococcal Invasive Disease by Inhibiting Bacterial Transmigration across the Lung

**DOI:** 10.1371/journal.ppat.1003727

**Published:** 2013-11-07

**Authors:** Kim S. LeMessurier, Hans Häcker, Liying Chi, Elaine Tuomanen, Vanessa Redecke

**Affiliations:** Department of Infectious Diseases, St. Jude Children's Research Hospital, Memphis, Tennessee, United States of America; University of California, San Francisco, United States of America

## Abstract

*Streptococcus pneumoniae* infection is a leading cause of bacterial pneumonia, sepsis and meningitis and is associated with high morbidity and mortality. Type I interferon (IFN-I), whose contribution to antiviral and intracellular bacterial immunity is well established, is also elicited during pneumococcal infection, yet its functional significance is not well defined. Here, we show that IFN-I plays an important role in the host defense against pneumococci by counteracting the transmigration of bacteria from the lung to the blood. Mice that lack the type I interferon receptor (*Ifnar1* ^−/−^) or mice that were treated with a neutralizing antibody against the type I interferon receptor, exhibited enhanced development of bacteremia following intranasal pneumococcal infection, while maintaining comparable bacterial numbers in the lung. In turn, treatment of mice with IFNβ or IFN-I-inducing synthetic double stranded RNA (poly(I:C)), dramatically reduced the development of bacteremia following intranasal infection with *S. pneumoniae*. IFNβ treatment led to upregulation of tight junction proteins and downregulation of the pneumococcal uptake receptor, platelet activating factor receptor (PAF receptor). In accordance with these findings, IFN-I reduced pneumococcal cell invasion and transmigration across epithelial and endothelial layers, and *Ifnar1* ^−/−^ mice showed overall enhanced lung permeability. As such, our data identify IFN-I as an important component of the host immune defense that regulates two possible mechanisms involved in pneumococcal invasion, i.e. PAF receptor-mediated transcytosis and tight junction-dependent pericellular migration, ultimately limiting progression from a site-restricted lung infection to invasive, lethal disease.

## Author Summary

*Streptococcus pneumoniae* infection is a leading cause of bacterial pneumonia and invasive diseases such as sepsis and meningitis, which are associated with high morbidity and mortality. Here we identified type I Interferons (IFN-I) as critical mediators that prevent the progression of a local lung infection with *S. pneumoniae* to invasive disease. We found that mice lacking the receptor for IFN-I, or which received antibodies that interfere with receptor activation, showed increased development of bacteremia upon lung infection with *S. pneumoniae*. Treating mice, or cell lines, with IFN-I protected against bacterial migration across epithelial and endothelial cell barriers, correlating with increased expression of tight junction proteins, which enhance the lung's barrier function, and reduced surface expression levels of platelet activating factor receptor, a host receptor known to be hijacked by bacteria for migration across the lung/blood and blood/brain barriers. Together, our results identify IFN-I as an important component of the host immune defense against invasion from a gram-positive, extracellular bacterium, possibly reflecting a general mechanism for the regulation of epithelial and endothelial barrier function that is critical for protection from pathogen invasion.

## Introduction

*S. pneumoniae* is a common commensal bacterium in the human nasopharynx, and persists asymptomatically in this niche until it is usually cleared by the host several weeks after acquisition [1]–[3]. However, under certain conditions, pneumococci can migrate from this niche into the lungs where it is an important causative agent of bacterial pneumonia, especially in the elderly and children under 5 years of age [4]. The lung epithelial layer is a significant barrier in pneumococcal pathogenesis, and its breach results in invasive disease, which is associated with high mortality and further complications such as the development of meningitis [5]–[7]. Although pneumococcal migration across epithelial and endothelial barriers is a precondition in the pathogenesis of invasive pneumococcal disease, the precise mechanisms and factors that promote or counter-regulate the crossing of epithelial or endothelial cell layers are still incompletely understood.

Two major mechanisms have been shown to govern pneumococcal migration across epithelial and endothelial barriers: Receptor-mediated epithelial endo- and transcytosis, and non-selective pericellular migration through the interruption of tight junctions [8]. The predominant receptor involved in epithelial endo- and transcytosis in the lower respiratory tract is the G-protein-coupled receptor platelet activating factor receptor (PAF receptor) [9]–[11], which can bind to phosphoryl-choline present in the pneumococcal cell wall. Pro-inflammatory cytokines such as IL-1β or TNFα, which are elicited during pneumococcal infection upregulate PAF receptor, which may contribute to pneumococcus binding. Following its binding, PAF receptor is internalized and recycled resulting in the transportation of the bacterium to the basolateral side of the epithelial cells ultimately leading to systemic dissemination [9]–[11]. Accordingly, PAF receptor-deficient mice or mice treated with PAF receptor antagonists are less sensitive to bacterial transmigration and progression to invasive disease upon lung infection with pneumococci [9], [11], [12]. In addition to receptor-mediated transcytosis, modulation of epithelial permeability and pericellular migration due to disruption of tight junctions upon pnemococcal infection has been reported [8], [13]. Pericellular migration was attributed, at least in part, to plasminogen/plasmin binding to pneumococcal receptors, thereby enhancing cell adhesion and enzymatic cleavage of tight junction proteins [14]

We found that IFN-I regulates both mechanisms, i.e. receptor-mediated transcytosis as well as pericellular migration of pneumococci across epithelial barriers. While the role of type I interferon (IFN-I) in viral infection is well established and includes inhibition of virus replication and activation of adaptive immune responses [15]–[18], the specific role of IFN-I during bacterial infections, in particular infections caused by extracellular bacteria such as *S. pneumoniae*, is less well characterized. The IFN-I family consists of multiple IFNα and a single IFNβ protein [19]. Both IFNα and IFNβ are recognized by a heterodimeric receptor composed of the two subunits IFNAR1 and IFNAR2 [20], and in general promote an anti-inflammatory response during infection, for example by reducing the production of pro-inflammatory cytokines IL-1 and TNFα [21], [22]. IFNβ mRNA has been reported to be substantially upregulated upon pneumococcal infection and during pneumococcal carriage [23]–[25] and mice lacking IFNAR1 or IFNβ display prolonged nasopharyngeal carriage and enhanced mortality upon pneumococcal infection [24], [26]. In macrophages, IFN-I production has been shown to depend on pneumococcal uptake and the presence of pneumolysin [25], [27], involving STING-dependent cytosolic recognition of bacterial DNA [27] and recognition of bacterial peptidoglycan through the pathogen recognition receptor NOD2 [25]. In contrast to macrophages, lung epithelial cells can produce IFN-I independent of pneumococcal internalization, but also require the recognition of intracellular bacterial DNA and the DAI/STING/TBK1/IRF3 signaling cascade [24].

Here, we show that pneumococcal infection leads to rapid upregulation of IFNβ in the lung, which we found to be critical for protection against the progression of a localized lung infection to invasive disease. We provide evidence that IFNβ upregulates tight junction proteins and downregulates PAF receptor expression *in vitro* and *in vivo*, which correlates with reduced bacterial invasion and transmigration of lung epithelial cells and endothelial cells *in vitro*. In accordance with these functions of IFN-I, intranasal (i.n.) administration of IFNβ was found to protect mice against the development of systemic disease following i.n. infection with pneumococci. Taken together, our data suggest a novel mechanism by which IFNβ exerts protection against invasion from a gram-positive, extracellular bacterium at the epithelial barrier.

## Results

### Ifnb1 mRNA is upregulated early in the lungs of mice intranasally infected with pneumococci

**Figure 1:**
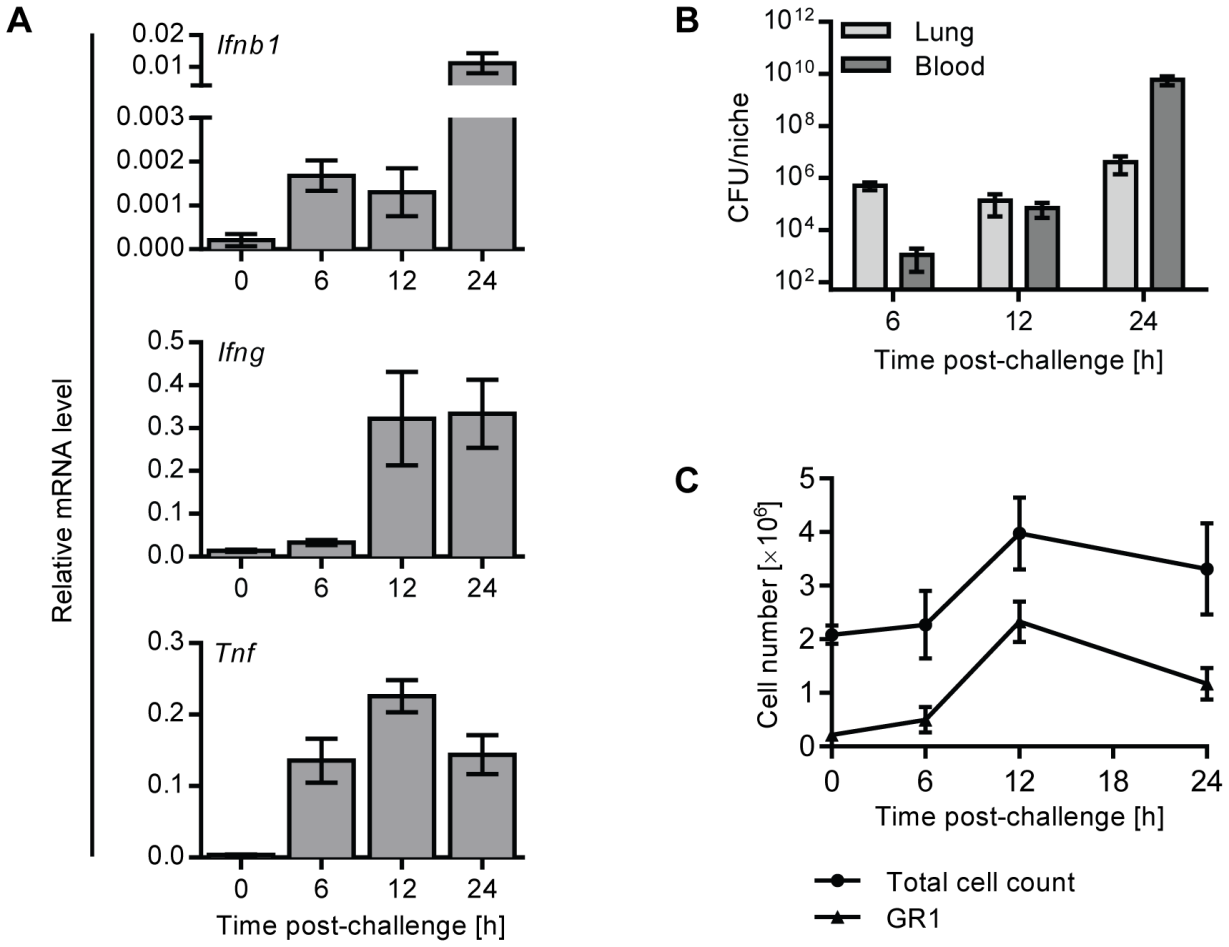
Expression of IFNβ is upregulated early in the lungs of mice upon intranasal pneumococcal infection. (A, B) 6 week old C57BL/6J mice were i.n. infected with 1×10^6^ CFU D39X in 50 µl PBS, or 50 µl PBS alone (0 h). (A) After 6, 12 and 24 hours, mRNA levels of *Ifnb1*, *Ifng* and *Tnf* were determined in whole lung homogenates by Q-PCR and normalized to *Cyclophilin* expression. n = 3–4. (B) Bacterial CFUs in the lungs were determined at indicated time points. Bars indicate mean value ± SEM (n = 3–4). (C) In separate experiments, mice were challenged as above (A, B) and lungs were perfused and collagenase-treated at various time points following infection. Total cell numbers were enumerated and expression of GR1 on live cells was analyzed by flow cytometry. Line indicates mean value ± SEM (n = 3–4).

To investigate the kinetics of IFNβ production during pneumococcal lung infection, we i.n. infected C57BL/6J mice with D39X, a stable bioluminescent isolate of the serotype 2 *S. pneumoniae* strain D39, which causes pneumonia and bacteremia in mice [28]. 6, 12 and 24 hours after infection, mRNA levels of *Ifnb1*, *Tnf* and *Ifng* were assessed in whole lung homogenates by Q-PCR, demonstrating an early upregulation of *Ifnb1* as soon as 6 hours after infection, which continued to rise at later time points. *Ifnb1* upregulation was accompanied by increasing levels of *Tnf* and *Ifng* mRNAs, reflecting the initiating host immune response (Figure 1A). Bacterial titers in the bloodstream were very low and close to the detection limit at 6 hours (Figure 1B), indicating that upregulation of *Ifnb1* mRNA reflected the acute local immune response, rather than secondary consequences of bacteremia. As there was very little immune cell infiltration of the lung at six hours post infection (Figure 1C), it seems likely that the primary IFN-I response is mediated by lung resident cells, such as alveolar macrophages or epithelial cells [24], [27], rather than recruited immune cells.

### Ifnar1^−/−^ mice show accelerated development of bacteremia following intranasal challenge with pneumococci

**Figure 2:**
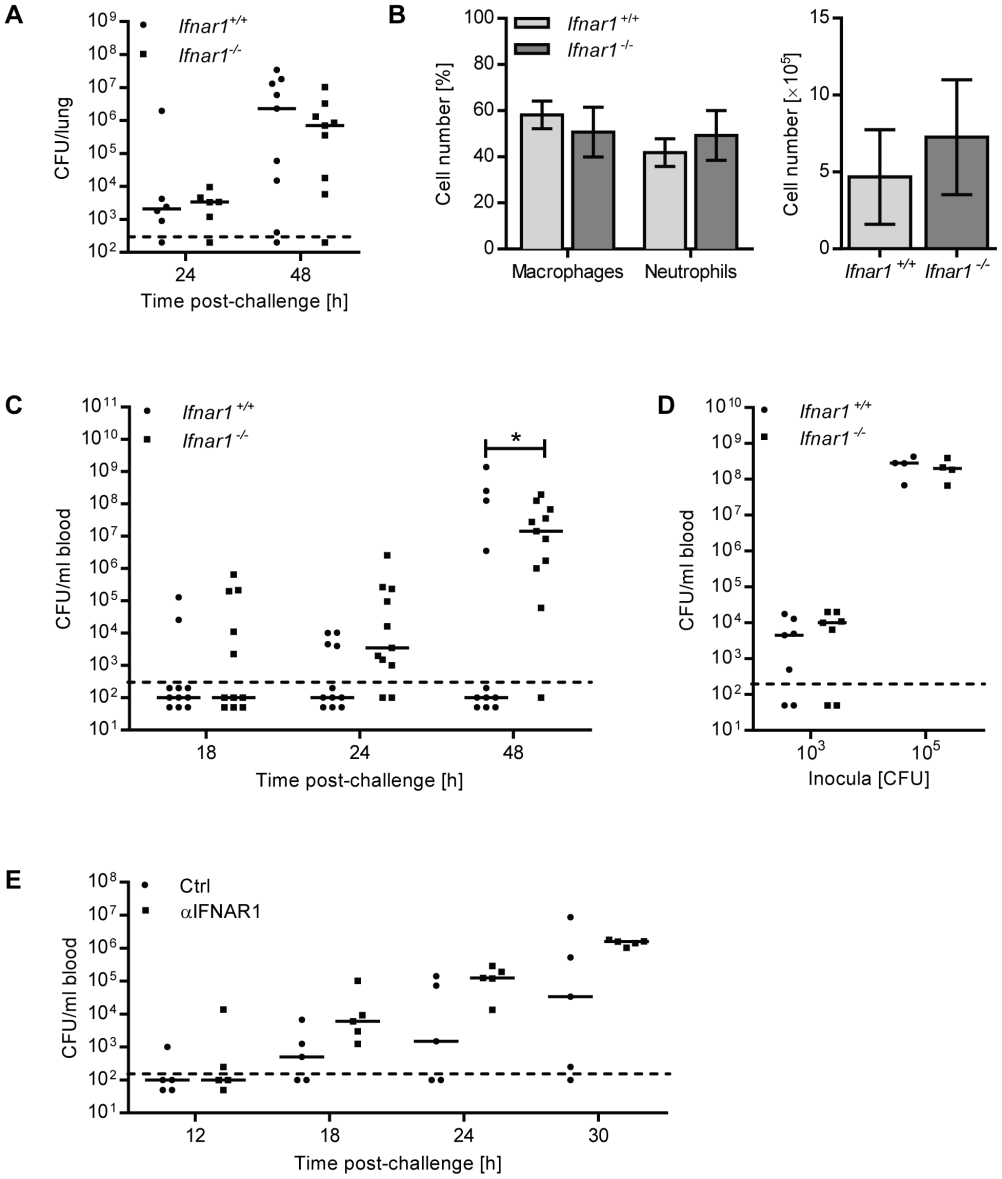
*Ifnar1^−/−^* mice and anti-IFNAR1treated mice are hypersusceptible to developing bacteremia. (A–C) 6 week old *Ifnar1^+/+^* and *Ifnar1^−/−^* mice were i.n. challenged with 4×10^5^ CFU D39X. (A) 24 and 48 hours after infection, mice were euthanized and pneumococcal CFU in whole lung homogenates were determined. 24 hours post-challenge n = 6, 48 hours post-challenge n = 9. Bars indicate median values. Dashed line indicates the limit of detection. (B) In a separate experiment, total cell numbers in the BAL and differential cell count were determined 24 hours after infection. Data represent mean ± SEM, n = 6. (C) Bacterial blood titers were determined at 18-, 24- and 48 hours post-challenge. Bars indicate median values * P<0.05 (Fisher Exact); n = 11. Dashed line indicates the limit of detection. (D) 6 week old *Ifnar1^+/+^* and *Ifnar1^−/−^* mice were i.p. infected with 1×10^3^ or 1×10^5^ CFU D39X. Bacterial titers in the blood and spleen were determined 12 hours after infection; n = 7 (1×10^3^ CFU), n = 4 (1×10^5^ CFU). (E) 6 week old C57Bl6/J mice were i.n. inoculated with 70 µg anti-IFNAR1 antibody or isotype control, and 1 hour later were i.n. challenged with 4×10^5^ CFU D39X and additional 30 µg anti-IFNAR1 antibody or isotype control. Bacterial titers in the blood were determined at 12-. 18-, 24- and 30 hours post-challenge. Bars indicate median values, n = 5. Dashed line indicates the limit of detection.

To elucidate the function of IFN-I during pneumococcal infection, we i.n. challenged *Ifnar1* ^−/−^ and *Ifnar1* ^+/+^ mice with D39X and followed disease progression. No significant differences in immune cell recruitment or bacterial titers in the lungs of *Ifnar1* ^−/−^ and *Ifnar1* ^+/+^ mice were observed after pneumococcal challenge (Figure 2A, B). However, in the peripheral blood, pneumococci were detected significantly earlier and at higher numbers in *Ifnar1* ^−/−^ mice compared to *Ifnar1* ^+/+^ animals (Figure 2C), with 45% of the *Ifnar1* ^−/−^ mice exhibiting bacteremia by 18 hours post-challenge compared to 18% of the *Ifnar1* ^+/+^ animals. This difference was maintained at 24 and 48 hours after infection, by which time 91% of the *Ifnar1* ^−/−^ mice were bacteremic compared to 36% of the *Ifnar1* ^+/+^ mice. In contrast to the intranasal challenge, intraperitoneal (i.p.) infection of *Ifnar1* ^−/−^ and *Ifnar1* ^+/+^ mice resulted in similar bacterial titers in the blood 12 hours post-infection (Figure 2D). Given the comparable numbers of pneumococci in the lung of *Ifnar1* ^+/+^ and *Ifnar1* ^−/−^ mice (Figure 2A) upon intranasal infection, these data suggested that IFN-I influences the transition of pneumococci from the lung into the bloodstream.

### Neutralizing IFNAR1 in the lung enhances pneumococcal migration to the bloodstream

To complement our studies based on IFNAR1-deficient mice, C57BL/6 mice were inoculated i.n. with a neutralizing antibody against IFNAR1, or isotype control. One hour later, mice were i.n. challenged with D39X in combination with a second dose of anti-IFNAR1 (or isotype control). Colony-forming units (CFU) in the bloodstream were determined at 12, 18, 24 and 30 hours after bacterial challenge. Mice that were treated with anti-IFNAR1 developed bacteremia faster than those that received the isotype control antibody (Figure 2E). The median blood bacterial titer was also increased by anti-IFNAR1 treatment: At 18 hours post-infection, the median bacterial titer was 11-fold higher in anti-IFNAR1-treated animals, a difference which further increased at 24 h and 30 h to 19- and 17-fold, respectively (Figure 2E). Although this did not reach statistical significance due to the limitation in mouse numbers feasible in this type of experiment, the data support the results obtained with *Ifnar1* ^−/−^ mice, suggesting that IFN-I activity during pneumococcal infection controls transition of pneumococci from the lung into the bloodstream.

### IFNβ pre-treatment protects against pneumococcal bacteremia following intranasal challenge

**Figure 3:**
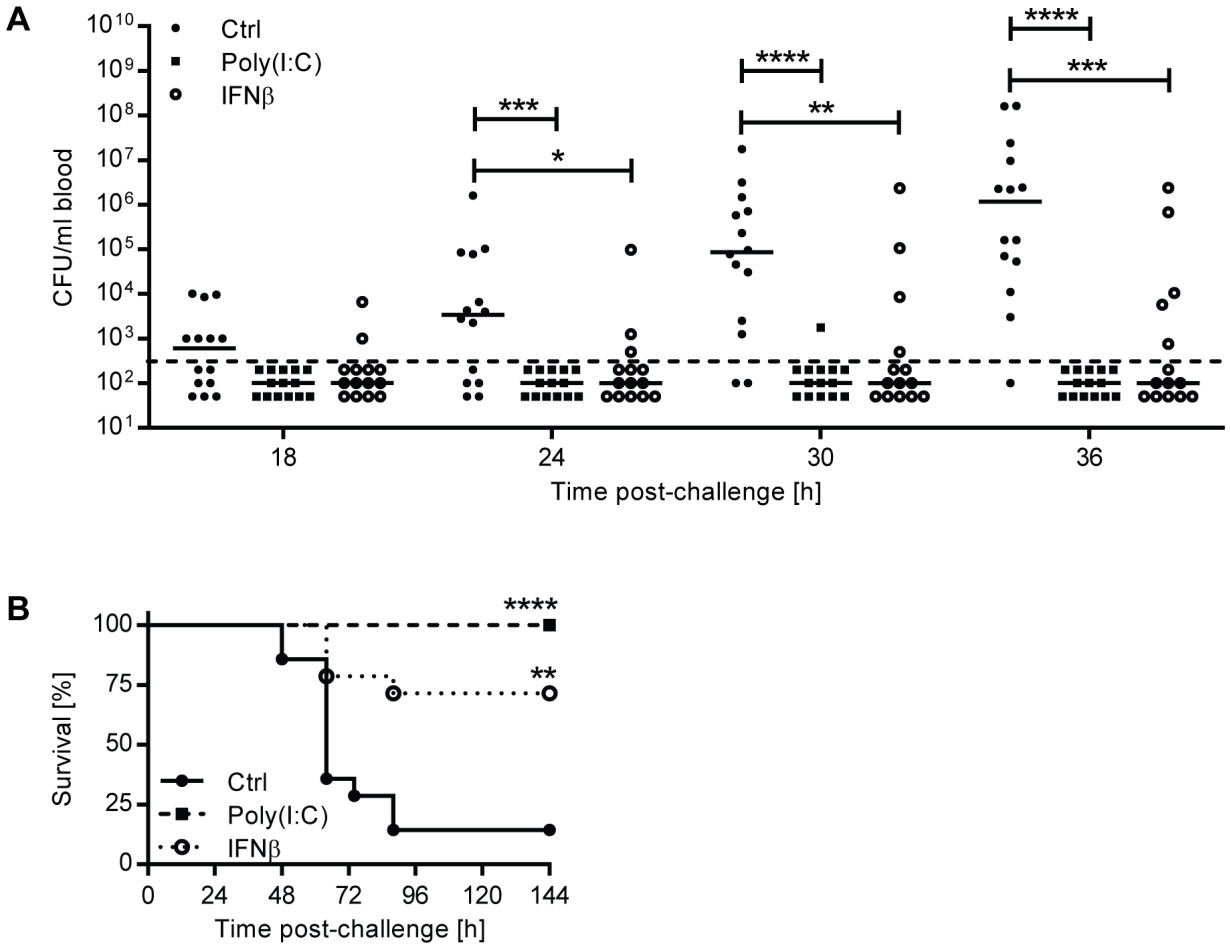
Pre-treatment of mice with IFNβ protects against the development of invasive disease. (A–B) 6 week old C57BL/6J mice were pretreated with recombinant mouse IFNβ, poly(I:C) or PBS (Ctrl) i.n. 24 hours before intranasal infection with 5×10^6^ CFU D39X. (A) Blood CFU titers were determined at various time points and analyzed using the Mann-Whitney test. Bars represent median, n = 14. Dashed line indicates the limit of detection. (B) Survival of mice was analyzed using the Log-Rank test. n = 14. For all graphs, ** P<0.01, *** P<0.005, **** P<0.001.

Since the loss of IFN-I activity in *Ifnar1^−/−^* mice led to increased susceptibility to bacteremia upon intranasal infection, we wished to determine whether prophylactic treatment of mice with recombinant IFNβ in turn might confer protection. To this end we treated mice i.n. with recombinant IFNβ or poly(I:C) (polyinosinic:polycytidylic acid), a synthetic analog of double-stranded RNA which induces endogenous IFN-I. 24 hours after treatment, mice were i.n. challenged with a lethal dose of pneumococci and bacterial titers in the blood were determined at different time points. The number of mice that developed bacteremia, and also the corresponding blood bacterial titers were significantly reduced upon IFNβ or poly(I:C) treatment (Figure 3A). This protective effect was observed as early as 18 hours post-challenge, when bacteremia was detectable in 36% of vehicle-treated mice versus 14% and 0% of IFNβ- and poly(I:C)-treated mice, respectively. Reduced bacteremia in IFNβ- and poly(I:C)-treated mice correlated with significantly increased survival (Figure 3B).

### IFNβ regulates lung permeability and expression of tight junction proteins

**Figure 4:**
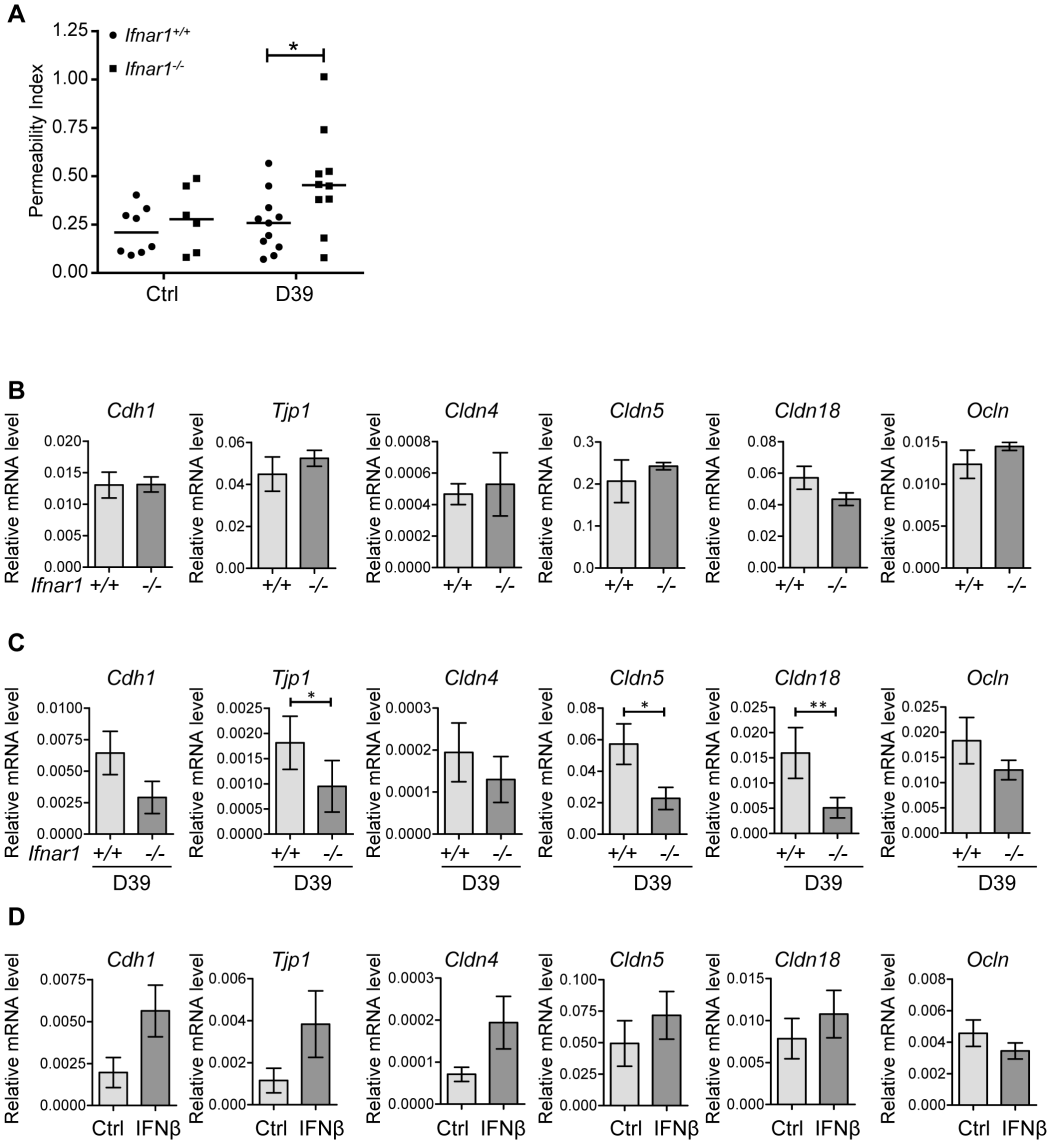
IFN-I decreases lung permeability and enhances expression of tight junction proteins. (A) 6 week old *Ifnar1^+/+^* and *Ifnar1^−/−^* mice were i.n. challenged with 4×10^5^ CFU D39X. 16 hours later lung permeability was determined by monitoring FITC-albumin leakage into the alveolar space, P<0.05. (B) mRNA levels of indicated genes were determined in the lungs of untreated *Ifnar1^+/+^* and *Ifnar1^−/−^* by Q-PCR (normalized to GAPDH expression), n = 5. (C) 6–8 week old *Ifnar1^+/+^* and *Ifnar1^−/−^* mice were i.n. challenged with 4×10^5^ CFU D39X. 24 hours after infection, mRNA levels of indicated genes were determined in the lungs of infected *Ifnar1^+/+^* and *Ifnar1^−/−^* by Q-PCR (normalized to GAPDH expression), n = 10. (D) 6 week old C57BL/6J mice were treated with recombinant mouse IFNβ and mRNA levels of indicated genes determined in the lungs after 24 hours, n = 6. Bars indicate mean ± SEM. * P<0.05, ** P<0.01 (Mann Whitney test).

Given the observation that IFN-I inhibited bacteremia without affecting the numbers of bacteria in the lung, we hypothesized that type I IFN may regulate the epithelial barrier function in the lung. We first investigated the overall lung permeability in infected and uninfected *Ifnar1^+/+^* and *Ifnar1^−/−^* mice by i.v. injection of FITC-labelled albumin, followed by analysis of FITC-albumin leakage into the alveolar space 16 hours after infection. *Ifnar1^−/−^* mice showed constitutively enhanced permeability of the lung which was significantly increased upon pneumococcal infection in comparison to *Ifnar1^+/+^* mice, the latter of which showed only a modest increase in lung permeability upon infection (Figure 4A). According to these observations, the mRNA levels of tight junction proteins, which guard the epithelial barrier functions, such as *Cdh1* (eCadherin), *Tjp1* (ZO-1), *Cldn4* (Claudin 4), *Cldn5* (Claudin 5) and *Cldn18* (Claudin 18) were found at lower levels in infected *Ifnar1^−/−^* mice (Figure 4C), while constitutive levels in uninfected animals were more comparable (Figure 4B). Moreover, intranasal treatment of mice with IFNβ resulted in increase of *Cdh1*, *Tjp1*, *Cldn4*, *Cldn5* and *Cldn18* (Figure 4D). Together, these data support the interpretation that IFN-I increases the resistance and integrity of the lung epithelial barrier during infection, thereby counteracting bacterial invasion. Of note, the effect of IFN-I on tight junction proteins displayed selectivity, as the expression levels of Occludin and other members of the Claudin family were not affected (Figure 4C, D and data not shown).

### IFNβ treatment of polarized respiratory epithelial cell layers counteracts pneumococcal transmigration

**Figure 5:**
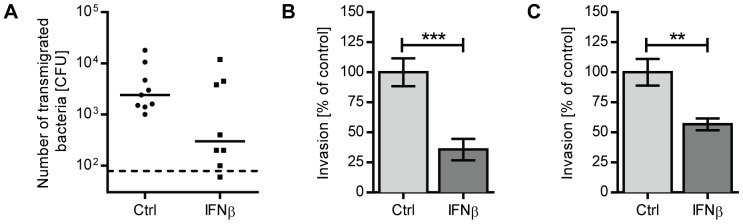
IFNβ reduces pneumococcal transmigration and invasion of lung epithelial cells and endothelial cell lines. (A) 16HBE cells were grown at an air-liquid interface in a transwell system until they reached a TER of at least 350 ohm cm^2^. Cells were treated with 1000 U/ml recombinant IFNβ or PBS for 20 hours. 2×10^4^ CFU R6 were added to the apical chamber, and bacteria in the basolateral chamber were enumerated after 10 hours. Three independent experiments are combined. n = 8 (IFNβ) and n = 9 (PBS). Bars indicate median CFU. Dashed line indicates the limit of detection. (B) A549 cells or (C) rBCEC_6_ cells were incubated with 1000 U/ml recombinant IFNβ or PBS for 20 hours prior to performing an invasion assay with R6. Data from three independent experiments are combined. For assays using A549 cells, n = 10 (PBS) and n = 9 (IFNβ). For rBCEC_6_ results, n = 7 (PBS) and n = 6 (IFNβ). Bars indicate mean ± SEM. ** P<0.01, *** P<0.005 (Mann Whitney test).

To test if IFN-I directly modulates epithelial barrier function, we used an in vitro transwell system to determine the migration of pneumococci through an IFNβ-treated layer of 16HBE14o- bronchial epithelial cells (16HBE cells), a cell line that can polarize and be sustained at confluence, thus mimicking the lung epithelial barrier *in vivo* [29]. As the pneumococcal capsule interferes with bacterial uptake in these *in vitro* settings [30], an unencapsulated derivative of D39, R6, was used. While IFNβ treatment did not affect epithelial polarization and cell confluence itself (as determined by transepithelial resistance (TER) measurements) or the total number of pneumococci recovered from the apical chamber during the course of the experiment (data not shown), the number of pneumococci that crossed the epithelial cell layer was significantly lower if cells had been treated with IFNβ (Figure 5A). Together, the *in vivo* and *in vitro* data suggests that IFN-I acts on lung epithelial cells to prevent pneumococcal transmigration and thus invasion into the bloodstream.

### IFNβ reduces pneumococcal invasion of lung epithelial cells and brain microvascular endothelial cells

In addition to the pericellular route controlled by tight junction proteins, pneumococci were also shown to transmigrate across epithelial or endothelial barriers by host cell adhesion, host cell invasion and transport of engulfed bacteria from the apical side to the basolateral side of the epithelial cell. One well-characterized mechanism that operates in the lower respiratory tract is based on the specific interaction between phosphoryl-choline moieties contained in the bacterial cell wall and PAF receptor expressed on host cells [9]–[11]. To determine whether IFN-I also interferes with cellular uptake of bacteria, we examined the capacity of R6 to invade A549 lung epithelial cells that had been incubated with recombinant IFNβ for 20 hours. Extracellular bacteria adhering to the cells were killed by antibiotic treatment prior to cell lysis. As shown in Figure 5B, pre-treatment of A549 cells with IFNβ significantly reduced uptake of pneumococci. IFNβ treatment neither affected the viability of A549 cells nor the viability of pneumococci (Figure S1A, C). Given that pneumococci utilize a similar mechanism to invade endothelial cells in order to cross the blood-brain barrier, we also examined the effect of IFNβ on bacterial uptake by the rat brain endothelial cell line, rBCEC_6_. Similar to A549 cells, IFNβ-treatment significantly reduced the number of bacterial uptake by rBCEC_6_ cells (Figure 5C). As such, IFNβ regulates pneumococci invasion of epithelial and endothelial cells, further contributing to the host resistance against bacterial transmigration.

### IFNβ decreases PAF receptor levels in epithelial and endothelial cells and in lungs in vivo

**Figure 6:**
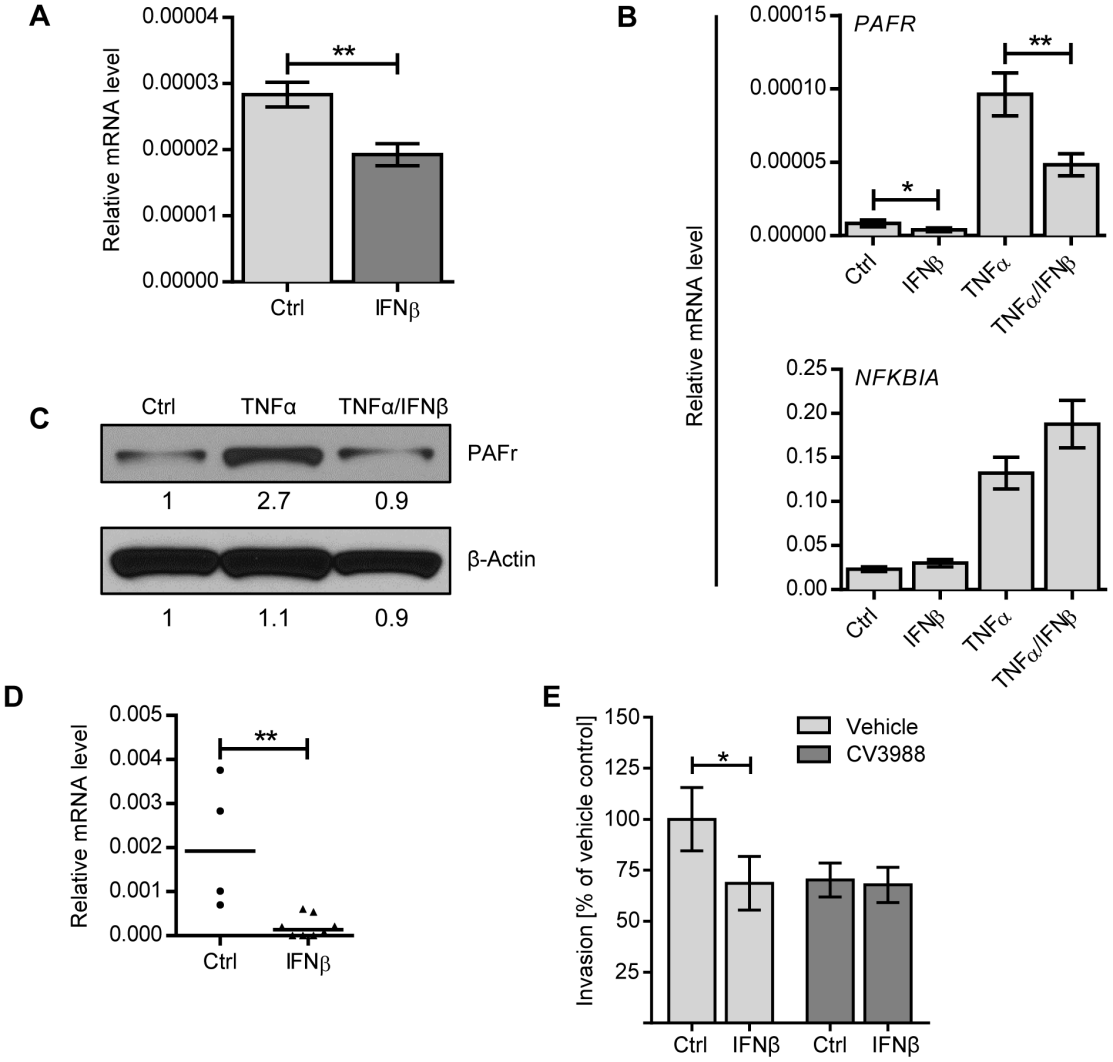
IFNβ reduces PAF receptor levels on lung epithelial and endothelial cell lines and in the lung. (A) A549 cells were stimulated with 1000 U/ml IFNβ, or an equal volume of PBS, for 8 h and PAF receptor mRNA levels were determined by Q-PCR (normalized to *β-ACTIN* expression), n = 9. (B) A549 cells were incubated with or without 1000 U/ml IFNβ, 10 ng/ml TNFα or both IFNβ and TNFα for 8 h and expression levels of *PAFR* and *NFKBIA* were determined by Q-PCR (normalized to *β-ACTIN* expression). n = 7–8. Statistical analysis was performed with the Mann-Whitney test. * P<0.05, ** P<0.01. Data shown are representative of three experiments. (C) rBCEC_6_ cells were stimulated with or without 10 ng/ml TNFα or TNFα in combination with 1000 U/ml IFNβ for 24 hours. Total lysates were analyzed by immuno-blotting using antibodies against PAF receptor and β-ACTIN. Relative protein expression levels were quantified by Image Studio Lite software. (D) C57BL/6 mice were treated i.n. with recombinant mouse IFNβ and euthanized after 24 hours. PAF receptor mRNA levels were determined by Q-PCR (normalized to cyclophilin expression). Bars represent median value, n = 4–8, ** P<0.01. (E) A549 cells were incubated with 1000 U/ml recombinant IFNβ or PBS for 20 hours and PAF receptor antagonist CV3988 or vehicle control for 30 minutes prior to performing an invasion assay with R6. Data from three independent experiments are combined. n = 12. Bars indicate mean ± SEM. * P<0.05 (Mann Whitney test).

As pneumococcal transmigration across epithelial and endothelial barriers involves receptor-mediated endocytosis mediated by PAF receptor, we investigated if the regulation of PAF receptor expression was affected by IFNβ treatment. Indeed, IFNβ-treatment of A549 cells led to significant downregulation of *PAFR* mRNA levels (Figure 6A). Furthermore, IFNβ treatment of A549 cells counteracted upregulation of *PAFR* mRNA induced by TNFα, an inflammatory cytokine known to increase PAF receptor expression (Figure 6B). In contrast, IFNβ treatment did not affect the expression of *NFKBIA*, which encodes IkBα, a well-characterized TNFα-induced NF-κB target gene (Figure 6B) [31]–[33], or the expression of other molecules described in context with pneumococcal adhesion and uptake, such as the polymeric immunoglobulin receptor, keratin10, laminin receptor, vitronectin and the complement component factor H [34]–[39], (Figure S2A). IFNβ-dependent effects on PAF receptor mRNA levels were also reflected on a protein level as determined by immuno-blot analysis of rBCEC_6_ cells that were treated with TNFα, either alone or in combination with IFNβ (Figure 6C). Moreover, intranasal IFNβ treatment of mice led to downregulation of *PAFR* mRNA levels in the lung (Figure 6D). In contrast and consistent with our in vitro findings, other molecules associated with pneumococcal adhesion and uptake were not affected by IFNβ in vivo-treatment (Figure S2B). To further confirm the correlation between IFNβ-mediated downregulation of PAF receptor and reduced bacterial uptake, we performed invasion assays in the presence of PAF receptor antagonist. As expected, the treatment of A549 cells with the PAF receptor antagonist alone reduced bacterial invasion. PAFR antagonist treatment did not affect the viability of A549 cells or the viability of pneumococci (Figure S1B, D). PAF receptor inhibition of IFNβ-treated cells did not result in further reduction of bacterial invasion (Figure 6E). These data are consistent with the interpretation that both, PAF receptor antagonist and IFNβ act via the same mechanism, i.e. the interference with PAF receptor -mediated bacterial uptake. These data demonstrate that IFNβ reduces constitutive and TNFα-induced expression of PAF receptor, which correlates with the decreased pneumococcal uptake by epithelial cells. Collectively, our results show that IFN-I contributes to two mechanisms counteracting pneumococcal invasion, i.e. first, the up-regulation of tight junction proteins controlling bacterial pericellular migration and second, the downregulation of PAF receptor, interfering with bacterial uptake and transmigration.

## Discussion

Here, we show that intranasal infection with *S. pneumoniae* results in early upregulation of IFN-I, which is critically involved in host protection from pneumococcal invasion. As demonstrated by loss-of-function and gain-of-function experiments, IFN-I signaling neither affected bacterial cell numbers in the lung, nor bacterial blood titers upon systemic infection, but reduced and delayed the appearance of pneumococci in the blood upon intranasal infection, indicating the barrier function of the lung as an IFN-I target.

Epithelial cells form a physical barrier against invading pathogens and are part of the first line of host defense in the lung against invading pathogens. While the epithelial barrier is relatively impermeable under normal circumstances, some pathogens have acquired mechanisms to penetrate this barrier leading to invasive disease [11], [40], [41]. Migration from the lung to the bloodstream can be increased by certain conditions, such as pneumonia [42]–[45], where host tissue injury and disruption of tight junctions or inflammation-dependent upregulation of host factors, such as PAF receptor expression in the case of *S. pneumoniae*, contribute to the development of systemic disease. Our data demonstrate a dual function of IFN-I on lung epithelial cells during pneumococcal infection: First, reduction of lung permeability due to stabilization of tight junction proteins and second, downregulation of PAF receptor expression resulting in reduced bacterial transmigration.

Several lines of evidence support the idea that IFN-I plays an important role for protection of the epithelial and endothelial barrier function. IFN-I has been shown to stabilize the permeability of the blood brain barrier and to block disintegration of the endothelial barrier induced by proinflammatory mediators, such as IFNγ, TNFα and histamine [46]–[48]. Also, TLR9-triggered protection against experimental colitis, which reflects bacterial invasion, was IFN-I dependent [49], and poly(I:C), a strong IFN-I inducer, protected mice against inflammatory bowel disease [50]. Furthermore, IFN-I treatment was shown to reduce host invasion by gram-negative bacteria, i.e. *Shigella spp*. and *Salmonella typhimurium* [51], [52]. All these data are consistent with IFN-I acting as a general protective factor of epithelial and endothelial barriers.

Notably, using other routes of infection, i.e. intravenous and intracranial pneumococcal challenge, previous reports have attributed the increased susceptibility of IFNAR1-deficient mice (or mice that had received an IFN-I neutralizing antibody) to reduced macrophage functions, such as nitric oxide and TNFα production [26], [53]. While it is not surprising to see that the pleiotropically active IFN-I impinges on various cell types, including innate immune cells, it is interesting to note that IFN-I did not affect bacterial clearance from the lungs upon intranasal challenge, nor did it affect bacterial clearance from the blood upon i.p. infection (Figure 2A and D). Similar results were reported by Shahangian *et al.*, who found no difference in pneumococcal titers in the lungs of *Ifnar1^+/+^* and *Ifnar1^−/−^* mice upon intratracheal infection [54]. Based on these data it appears that IFN-I controls different, tissue-specific functions which collectively counteract bacterial invasion and multiplication during primary *S. pneumoniae* infection.

*Ifnb1* mRNA in the lung was found to be upregulated before the onset of bacteremia, indicating its activation by the acute local immune response rather than secondary factors associated with bacteremia. The primary source of IFN-I in the lung upon pneumococcal infection is still unclear. Macrophages, dendritic cells and epithelial cells have been found to produce IFN-I upon pneumococcal stimulation [24], [27]. Depletion of alveolar macrophages (AM) by administration of liposomal Cl2MBP before intranasal infection with *S. pneumoniae* led to an increased local inflammatory response and enhanced mortality while the bacterial load in lung and blood was not affected [55]. While these observations are discrepant from those reported here for IFNAR1-deficient mice and therefore argue against a major role of AM as the IFN-I producing cells type, it should be noted that AM depletion resulted also in strongly increased granulocyte recruitment, making direct comparisons of the two situations difficult. As such, the critical, local source of IFN-I in the lung during pneumococcal infection still needs to be defined.

As shown here, IFNβ downregulates constitutive and TNFα-induced PAF receptor expression on lung epithelial cells, which correlates to reduced bacterial uptake and transmigration. Notably, IFNβ-mediated downregulation of PAF receptor was not limited to epithelial cells, but was also found on brain microvascular endothelial cells. Given that PAF receptor-deficient mice exhibit protection against the development of meningitis [11], our data indicate the possibility of a similar, protective function of IFNβ against bacterial transgression at the blood-brain barrier. Also, it is interesting to note that phosphoryl-choline is not restricted to the pneumococcal cell wall, but represents a common cell wall component of many pathogens [56]. Accordingly, PAF receptor was found to be involved in the translocation of gram-positive *Enterococcus faecalis* across intestinal epithelia [57]; it is thus tempting to speculate that IFNβ-mediated regulation of PAF receptor may represent a general protective mechanism against bacterial translocation across epithelial and endothelial barriers.

Apart from bacterial transmigration, PAF receptor is suspected to play a significant role in a variety of pathophysiological situations, including tumor biology, atherosclerosis and inflammatory diseases [58]–[60]. PAF receptor is widely expressed on immune and non-immune cells and its natural ligand, the phospholipid PAF, is a potent activator of inflammatory and thrombotic processes [60]. PAF activity is tightly regulated at different levels, including synthesis and degradation, and deregulated PAF signaling was found to contribute to the development of inflammatory and thrombotic disease [58]–[60]. Given the immuno-suppressive function of IFN-I, which is therapeutically used in Multiple Sclerosis, it is tempting to speculate IFN-I-mediated downregulation of PAF receptor may be involved in this process.

It is very interesting to note that PAF receptor activation via PAF has been shown to increase epithelial permeability by modifying expression levels of tight junction proteins [61], [62]. Thus, it is possible that IFN-I modulates the expression of tight junction proteins via limiting PAF receptor expression. In this scenario, IFN-I mediated downregulation of PAF receptor serves two functions, i.e. preventing systemic dissemination of pneumococci by reducing PAF receptor-mediated invasion of lung epithelial cells and inhibiting pericellular migration. However, from the pathogen perspective, PAF receptor could also play a dual function, i.e. serving as the uptake receptor and, via activation of its signaling function, as measure to downregulate tight junctions which results in a secondary loss of epithelial barrier integrity, allowing bacterial invasion. Certainly, whether pneumococci trigger those PAF receptor-mediated signaling pathways that control expression of tight junctions needs to be investigated.

IFN-I was reported to contribute adversely to a lethal pneumococcal challenge following infection with Influenza A virus, and also increased pneumococcal colonization of the nasopharynx during Influenza A co-infection [25], [54]. The reduced susceptibility of *Ifnar1^−/−^* mice in these co-infection models was attributed to impaired neutrophil and macrophage functions, such as reduced KC and MIP2 expression, in the presence of IFN-I. Consistent with our data (Figure 2A), no difference in pneumococcal clearance of wildtype and IFNAR1-deficient mice were detected, unless co-infected with Influenza A virus [54]. Somewhat surprising, we found that poly(I:C) treatment, which mimics certain aspects of viral infection, including IFN-I activation, resulted in protection against invasive disease. One possibility to explain these observations is that additional factors induced by Influenza A viruses contribute to the adverse effects of IFN-I observed in the co-infection model. Alternatively, the different kinetics of IFN-I production in the two models may be an important factor. While protective in the acute situation, as used in our model, IFN-I may lead to reduced bacterial resistance at later time points due to immuno-suppressive effects. Consistent with more general differences between mono- and co-infection models, PAF receptor-deficient mice were more resistant to pneumococcal disease, as mentioned, while less significantly protected upon Influenza A co-infection [63]. As such, a beneficial function of IFN-I during pneumococcal challenge may depend on the context of infection, which will also deserve attention in clinical situations and therapeutic settings.

## Materials and Methods

### Ethics statement

All animal studies were conducted under protocols approved by the St. Jude Children's Research Hospital Institutional Animal Care and Use Committee. The St Jude animal care program is fully accredited by the Association for the Assessment and Accreditation of Laboratory Animal Care, International (AAALAC-I). Laboratory animals are maintained in accordance with the applicable portions of the Animal Welfare Act and the guidelines prescribed in the DHHS publication, Guide for the Care and Use of Laboratory Animals.

### Bacterial strains and growth conditions

*S. pneumoniae* was routinely grown on tryptic soy agar (Difco Laboratories, Detroit, MI) supplemented with 3% sheep blood or in defined semisynthetic casein liquid medium supplemented with 0.5% yeast extract [64] at 37°C with 5% CO_2_. The following bacterial strains were used: *S. pneumoniae* serotype 2 strain D39 Xen7 (D39X) [28], a stable bioluminescent derivative of strain D39; R6, an unencapsulated variant of D39.

### Cell lines and culture conditions

A549 cells (ATCC) were grown in F12K media (Invitrogen) supplemented with 10% fetal bovine serum (GemCell), 2 mM L-glutamine (Cellgro), 100 U/ml penicillin and 100 U/ml streptomycin. The rat brain capillary endothelial cell line rBCEC_6_ [65] was cultured in Dulbecco's modified Eagle medium 4.5 g/ml glucose media (Invitrogen) supplemented with 10% fetal bovine serum, 2 mM L-glutamine, 110 µg/ml sodium pyruvate (Sigma), 8 µg/ml endothelial cell growth factor (Biomedical Technologies, Inc.), 80 µg/ml heparin. Transformed human bronchial epithelial 16HBE14o- cells (abbreviated as 16HBE) were kindly provided by Prof. Dieter C. Gruenert (University of California, San Francisco) and were grown in minimum essential medium with Earle's salt (Invitrogen) supplemented with 10% fetal bovine serum, 2 mM L-glutamine, 100 U/ml penicillin and 100 U/ml streptomycin. For bacterial migration assays, 16HBE cells were grown in transwell chambers (Corning, 24-mm, 3-µm pore size) as liquid-liquid interface cultures for at least 9 days, until a transepithelial resistance (TER) of at least 350 Ohm/cm^2^ was reached.

### Infection of mice

C57BL/6J mice were purchased from the Jackson Laboratory. B6.129S2-*Ifnar1^tm1Agt^*/Mmjax (*Ifnar1* ^−/+^) [66] mice were purchased from Mutant Mouse Regional Resource Centers and subsequently bred at the St. Jude Children's Research Hospital animal facility. For the *Ifnar1^−/−^*experiments, *Ifnar1^+/+^* littermate controls were used. Mice used in pneumococcal infection experiments were 5- to 6-week-old males. For infections, mice were lightly anesthetized with isoflurane and bacteria in 30 µl PBS (unless stated otherwise) were delivered to the nares. Mice were suspended vertically until they regained consciousness. The dose was confirmed by serial dilution of the inocula and growth on blood agar plates. For survival experiments, mice were monitored for a moribund state every 12 h until the termination of the experiment.

### Flow cytometry analysis

Single-cell suspensions of lungs were prepared by instilling 500 µl of collagenase type 2 (300 µg/ml, Worthington) in PBS into the trachea of mice. Lungs were excised, cut into small pieces and incubated in 1 mg/ml collagenase at 37°C with 5% CO_2_ for 20 min and then passed through a 70 micron cell filter. Red blood cells were lysed using ACK buffer (Lonza). Lung cells were blocked with Abs against CD16/CD32 (eBioscience), and stained for surface expression of GR1 (Ly6G, RB6-8C5, eBioscience). Flow cytometry data were acquired on a FACSCalibur flow cytometer (BD Biosciences), and data were analyzed using FlowJo software (Tree Star).

### BAL and differential cell count

The total numbers of cells in BAL were counted, and BAL cells were stained with Wright-Giemsa stain for the differential count. Percentages of neutrophils, and macrophages were determined microscopically.

### *In vivo* neutralization of IFNAR1

Mice were anaesthetized with isoflurane and i.n. inoculated with 70 µg anti-mouse IFNAR1 antibody (clone MAR1-5A3, low endotoxin functional grade, Leinco Technologies) or isotype control, in a volume of 70 µl. Mice were allowed to regain consciousness. After 1 h, mice were anaesthetized with isoflurane and i.n. challenged with pneumococci suspended in 30 µl PBS containing 30 µg anti-IFNAR1 or isotype control.

### IFNβ and poly(I:C) mouse treatment

Where specified, mice were lightly anesthetized with isoflurane and inoculated intranasally with 50 µl PBS containing 50 µg poly(I:C) (Invitrogen) or 30,000 U carrier-free mouse IFNβ (PBL InterferonSource) 24 h before intranasal challenge with *S. pneumoniae*. Control animals received an equivalent volume of PBS prior to bacterial challenge.

### Lung permeability studies

Lung permeability was determined by monitoring FITC-albumin distribution in bloodstream and bronchial space, as described previously [67]. Briefly, *Ifnar1+/+* or *Ifnar1−/−* mice were anaesthetized with isoflurane and 4×10^5^ CFU D39 were delivered to the nares. Control mice received 30 µl PBS intranasally. 100 µl of FITC-albumin (1 mg/ml, Sigma-Aldrich) in PBS was injected into the tail vein of mice 15 h post-challenge, and mice were euthanized by CO_2_ asphyxiation 1 h later. Bronchial alveolar lavages were performed using two consecutive washes with the same 1 ml PBS, and cells were removed by centrifugation at 400 g for 10 min. Blood was taken directly from the heart, and serum was recovered by centrifugation at 6000 g for 10 min. Fluorescence in serial dilutions of serum and BAL fluid (BALF) was determined at 486 nm, and is presented as a ratio of fluorescence in BALF to fluorescence in serum (Lung Permeability Index) for each mouse.

### Pneumococcal invasion assay

Pneumococcal invasion assays were performed as previously described [68]. Briefly, A549 cells were seeded in 24-well plates at a density of 8×10^4^ cells/500 µl/well and grown at 37°C in 5% CO_2_ for 24 h, after which they reached approximately 90% confluence. Where indicated, 1000 U/ml IFNβ was added to the media 20 h before the addition of bacteria. Pneumococcal cultures were grown to mid-log phase, washed, and then added to each well at 1×10^7^ CFU/well. Cells were incubated with the bacteria for 2 h, washed two times in dPBS (with magnesium and calcium), and incubated with penicillin (10 µg/ml) and gentamicin (200 µg/ml) in media for 1 h to kill extracellular bacteria. The cells were washed, trypsinized, and lyzed with 0.05% Triton X-100. Bacterial colonies were enumerated following overnight incubation on blood agar plates. For the PAF receptor antagonist studies, 10 µM CV3988 rac-3-(N-Octadecylcarbamoyl)-2-Methoxy) propyl-(2-thiazolioethyl) phosphate (CAS NO: 85703-73-7) (Enzo Life Science), or ethanol vehicle control, was added 30 minutes before infection [69].

### A549 viability assay

A549 cells were seeded in 24-well plates at a density of 8×10^4^ cells/500 µl/well and were either incubated with 1000 U/ml IFNβ or PBS as control for 24 h, or with 10 µM CV3988 or ethanol vehicle control for 2.5 hours. Cells were stained for AnnexinV and 7-AAD (Invitrogen) and cell viability was determined by flow cytometry.

### Pneumococcal viability assay

R6 was suspended in F12K media supplemented with 10% fetal bovine serum and 2 mM L-glutamine. Pneumococci were cultured at 37°C with 5% CO_2_, in the presence of 1000 U/ml human IFNβ, or 10 µM CV3988, or an equivalent volume of the corresponding vehicle control (PBS or ethanol, respectively). Bacterial growth was determined by enumeration of colony forming units on blood agar plates.

### *In vitro* bacterial migration assay

*S. pneumoniae* was grown to mid-log phase and diluted in tissue culture media (Dulbecco's modified Eagle's medium, 10% fetal bovine serum) without antibiotics. The media on the apical side of the transwell inserts was replaced with 1 ml bacterial suspension containing 2×10^4^ CFU R6, and the basolateral chamber was replaced with 2 ml media. After 10 hours, 20 µl of media was removed from the basolateral chamber and plated on blood agar plates. To investigate the effect of IFNβ on the migration of pneumococci across 16HBE cells, 1000 U/ml IFNβ was added to the apical chamber 20 h before the addition of bacteria.

### Quantitative real-time PCR

Total RNA was isolated from cells using Trizol (Sigma-Aldrich). For experiments where bacteria might be present, such as cells recovered from infected mice, cells were centrifuged at 300× g for 10 min and supernatant was discarded prior to RNA extraction, to minimize bacterial numbers. Nucleic acids were treated with DNase I Amplification Grade (Invitrogen) to remove contaminating DNA. The RNA was reverse transcribed to cDNA using the Superscript III first-strand cDNA synthesis kit (Invitrogen). Quantitative real-time PCR (Q-PCR) was performed on an AB 7300 real-time PCR machine (Applied Biosystems) using a SYBR green PCR Master Mix (Applied Biosystems). Each mRNA signal was normalized to cyclophilin (Cph) or β-actin (ActB) as a housekeeping gene. The following primer sequences against mouse genes were used: *Cph* sense, 5′-ATG GTC AAC CCC ACC GTG T; *Cph* antisense, 5′-TTC TTG CTG TCT TGG AAC TTT GTC; *Ifng* sense, 5′-CAT TCA GAG CTG CAG TGA CC-3′; *Ifng* antisense, 5′-CAC ATT CGA GTG CTG TCT GG-3′; *Ifnb1* sense 5′-AGC TCC AAG AAA GGA CGA ACA T-3′; *Ifnb1* antisense, 5′-GCC CTG TAG GTG AGG TTG ATC T-3′; *Pafr* sense, 5′-CTG GAC CCT AGC AGA GTT GG-3′ [70]; *Pafr* antisense, 5′-GCT ACT GCG CAT GCT GTA AA-3′ [70]; Tnf sense, 5′-ACA GAA AGC ATG ATC CGC G-3′; Tnf antisense, 5′-GCC CCC CAT CTT TTG GG-3′. *Cdh1* sense 5′- ACT GTG AAG GGA CGG TCA AC-3′; antisense 5′- GGA GCA GCA GGA TCA GAA-3′ [71]; *Tjp1* sense 5′-CGA GGC ATC ATC CCT AAT AAG AAC-3′; antisense 5′-TCC AGA AGT CTG CCC GAT CAC-3′ [72]; *Cldn5* sense 5′-ACG GGA GGA GCG CTT TAC-3′; antisense 5′-GTT GGC GAA CCA GCA GAG-3′; *Cldn4* sense 5′-ACG GGA GGA GCG CTT TAC-3′; antisense 5′-GAG CGC ACA ACT CAG GAT G-3′; *Cldn18* sense 5′-CCG GCC ATA CTT CAC CAT-3′; antisense 5′-CAT CAG GGC TCG TAC AGC TT-3′ [73].

The following primer sequences against human genes were used: ACTB sense, 5′-GAT CAT TGC TCC TCC TGA GC-3′; ACTB antisense, 5′- CGT CAT ACT CCT GCT TGC TG-3′; PAFR sense, 5′-TCT GCC TCA GCC TCT TTG TC-3′; PAFR antisense, 5′-ATG CTG TAA ACA ATC GGG AAG-3′.

### Western blotting

Cells were lyzed in XT Sample buffer (Biorad), cell lysates resolved by SDS-PAGE (Bio-Rad) and transferred onto nitrocellulose membranes. Membranes were probed with antibodies against PAF receptor (rabbit polyclonal, Cayman Chemical, 1∶1000) and β-Actin (clone AC-15, Sigma, 1∶10,000) and visualized using enhanced chemiluminescence (Pierce) for detection. Relative protein expression levels were quantified by Image Studio Lite software (Li-COR Biosciences).

### Statistical analysis

The Fisher Exact test was used to determine the significance of mice exhibiting bacteremia. Numbers of bacteria in the organs were compared between groups using the Mann-Whitney test. The statistical significance of survival duration observed for different mouse groups was analyzed using the log-rank test. P values of 0.05 or less were considered significant. The Fisher Exact test, log-rank test and Mann-Whitney test were performed using Prism version 5.03 (GraphPad Software, San Diego, CA).

## Supporting Information

Figure S1 **Cellular and bacterial viability upon IFNβ or PAFR antagonist treatment.** (A, B) A549 cells were incubated with 1000 U/ml recombinant IFNβ or PBS for 24 hours (A) or PAF receptor antagonist CV3988 or vehicle control for 2.5 hours (B). Cell viability was determined by flow cytometry. n = 3. Bars indicate mean ± SEM. (C, D) Pneumococcal strain R6 was cultured in F12K media supplemented with 10% fetal bovine serum and 2 mM L-glutamine (A549 tissue culture media) in the presence of 1000 U/ml recombinant IFNβ or PBS (C) or PAF receptor antagonist CV3988 or vehicle control (D) for 2 hours. Bacterial numbers were determined by colony formation on blood agar plates. n = 6. Bars indicate mean ± SEM.
(TIF)

Figure S2 **Expression of genes involved in pneumococcal adhesion and uptake upon IFN-I treatment.** (A) A549 cells were incubated with or without 1000 U/ml IFNβ, 10 ng/ml TNFα or both, IFNβ and TNFα, for 8 h and expression levels of indicated genes were determined by Q-PCR (normalized to GAPDH expression). Bars indicate mean ± SEM, n = 11.(B) C57BL/6 mice were treated i.n. with recombinant IFNβ. After 24 hours, expression levels of indicated genes were determined by Q-PCR (normalized to GAPDH expression). Bars indicate mean ± SEM, n = 6. *Complement component factor H (CFH, Cfh); laminin receptor (RPSA, Rpsa); Vitronectin (VTN, Vtn); keratin 10 (KRT10, Krt10); polymeric immunoglobulin factor receptor (PIGR, Pigr)*.
(TIF)
