# Supplementary figures and images for: Type I Interferon Protects against Pneumococcal Invasive Disease by Inhibiting Bacterial Transmigration across the Lung
Source: PLoS Pathog. 2013 Nov 7;9(11):e1003727. doi: 10.1371/journal.ppat.1003727 (PMC3820719; doi:10.1371/journal.ppat.1003727)

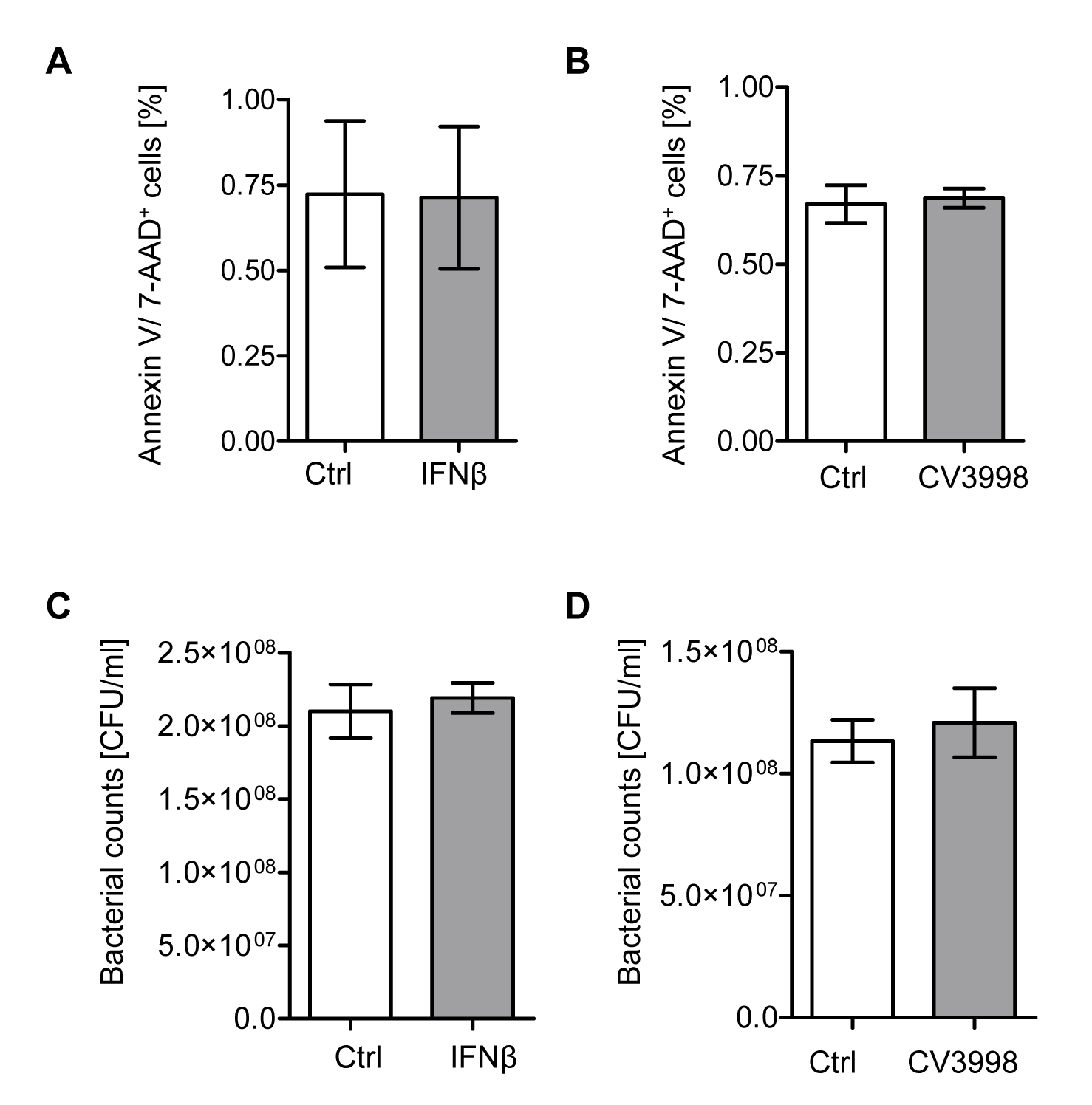

Supplement: Figure S1 — Cellular and bacterial viability upon IFNβ or PAFR antagonist treatment. (A, B) A549 cells were incubated with 1000 U/ml recombinant IFNβ or PBS for 24 hours (A) or PAF receptor antagonist CV3988 or vehicle control for 2.5 hours (B). Cell viability was determined by flow cytometry. n = 3. Bars indicate mean ± SEM. (C, D) Pneumococcal strain R6 was cultured in F12K media supplemented with 10% fetal bovine serum and 2 mM L-glutamine (A549 tissue culture media) in the presence of 1000 U/ml recombinant IFNβ or PBS (C) or PAF receptor antagonist CV3988 or vehicle control (D) for 2 hours. Bacterial numbers were determined by colony formation on blood agar plates. n = 6. Bars indicate mean ± SEM. (TIF) [file ppat.1003727.s001.tif]

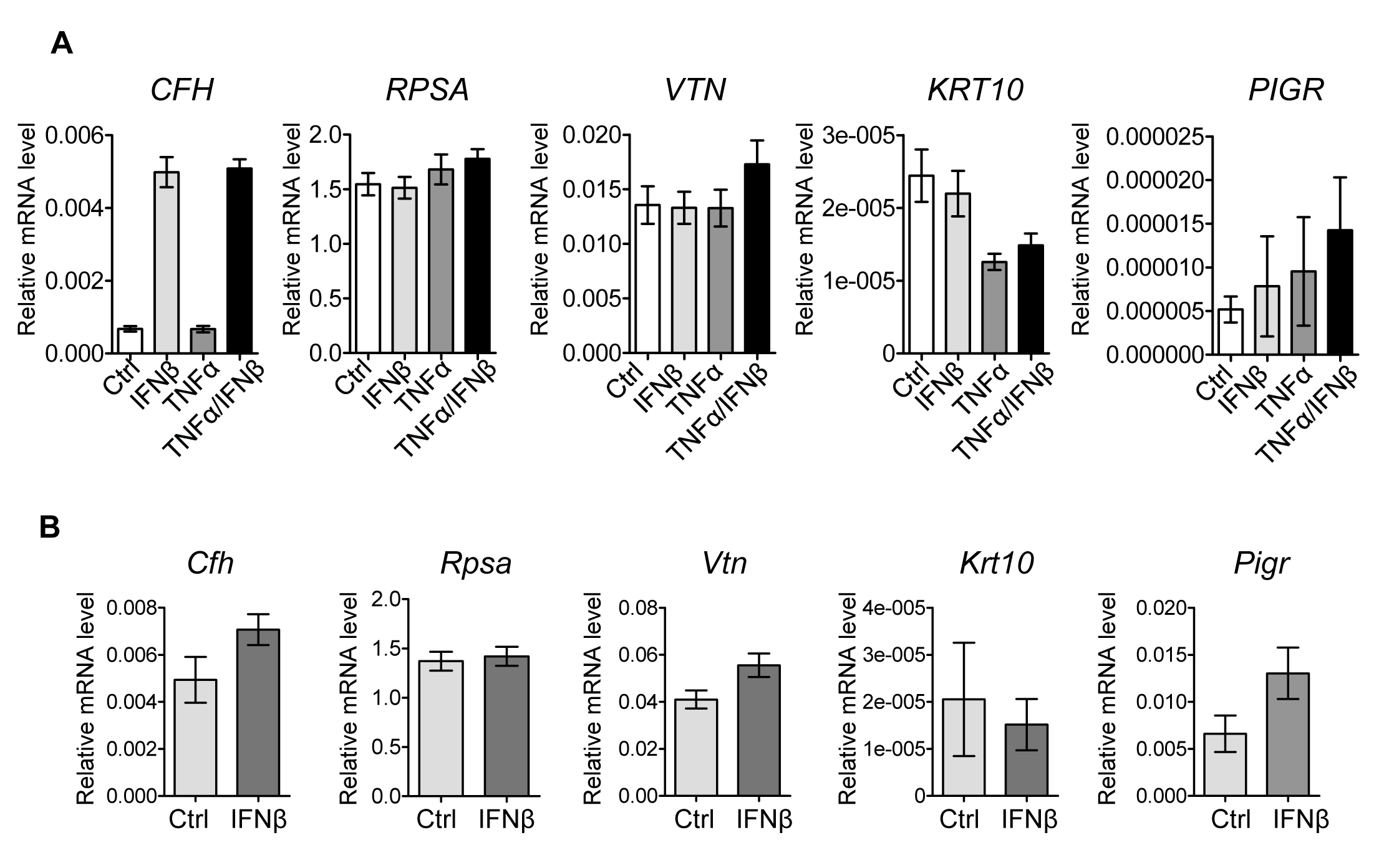

Supplement: Figure S2 — Expression of genes involved in pneumococcal adhesion and uptake upon IFN-I treatment. (A) A549 cells were incubated with or without 1000 U/ml IFNβ, 10 ng/ml TNFα or both, IFNβ and TNFα, for 8 h and expression levels of indicated genes were determined by Q-PCR (normalized to GAPDH expression). Bars indicate mean ± SEM, n = 11.(B) C57BL/6 mice were treated i.n. with recombinant IFNβ. After 24 hours, expression levels of indicated genes were determined by Q-PCR (normalized to GAPDH expression). Bars indicate mean ± SEM, n = 6. Complement component factor H (CFH, Cfh); laminin receptor (RPSA, Rpsa); Vitronectin (VTN, Vtn); keratin 10 (KRT10, Krt10); polymeric immunoglobulin factor receptor (PIGR, Pigr). (TIF) [file ppat.1003727.s002.tif]
